# Ru(II)-Thymine Complex Causes Cell Growth Inhibition and Induction of Caspase-Mediated Apoptosis in Human Promyelocytic Leukemia HL-60 Cells

**DOI:** 10.3390/ijms19061609

**Published:** 2018-05-30

**Authors:** Maiara de Souza Oliveira, Ádila Angélica Dantas de Santana, Rodrigo S. Correa, Milena Botelho Pereira Soares, Alzir Azevedo Batista, Daniel Pereira Bezerra

**Affiliations:** 1 Gonçalo Moniz Institute, Oswaldo Cruz Foundation (IGM-FIOCRUZ/BA), Rua Waldemar Falcão, 121, Candeal, Salvador 40296-710, Bahia, Brazil; msoliveira87@hotmail.com (M.d.S.O.); dantas.adila@gmail.com (A.A.D.d.S.); milenabpsoares@gmail.com (M.B.P.S.); 2 Department of Chemistry, Federal University of Ouro Preto, Ouro Preto 35400-000, Minas Gerais, Brazil; rodrigoquimic@gmail.com; 3 Center of Biotechnology and Cell Therapy, Hospital São Rafael, Salvador 41253-190, Bahia, Brazil; 4 Department of Chemistry, Federal University of São Carlos, São Carlos 13561-901, São Paulo, Brazil; daab@ufscar.br

**Keywords:** ruthenium, thymine, cytotoxicity, apoptosis, HL-60

## Abstract

Ruthenium-based compounds represent a class of potential antineoplastic drugs. Recently, we designed, synthesized, and identified the Ru(II)-thymine complex [Ru(PPh_3_)_2_(Thy)(bipy)]PF_6_ (where PPh = triphenylphosphine, Thy = thymine and bipy = 2,2′-bipyridine) as a potent cytotoxic agent with the ability to bind to DNA and human and bovine serum albumins. In this study, the underlying cytotoxic mechanism of the [Ru(PPh_3_)_2_(Thy)(bipy)]PF_6_ complex was assessed. This complex displayed potent cytotoxicity in different cancer cell lines; the morphology that is associated with apoptotic cell death, increased internucleosomal DNA fragmentation without cell membrane permeability, loss of the mitochondrial transmembrane potential, increased phosphatidylserine externalization, and caspase-3 activation were observed in human promyelocytic leukemia HL-60 cells that were treated with the complex. Moreover, pretreatment of HL-60 cells with Z-VAD(OMe)-FMK, a pan-caspase inhibitor, partially reduced the apoptosis that was induced by the complex, indicating that the apoptotic cell death occurred through a caspase-mediated pathway. In conclusion, the [Ru(PPh_3_)_2_(Thy)(bipy)]PF_6_ complex displays potent cytotoxicity to different cancer cells and induces caspase-mediated apoptosis in HL-60 cells.

## 1. Introduction

Cancer is a great public health problem in both developed and developing countries. In 2012, the estimate was approximately 14.1 million new cancer cases and 8.2 million deaths worldwide; among cancer, leukemia has one of the highest incidences worldwide, with approximately 352,000 new cases being diagnosed and approximately 265,500 deaths [1]. Chemotherapy remains an important therapy modality; however, due to side effects and drug resistance, new anticancer compounds are needed.

Ruthenium-based compounds form a class of potential antineoplastic drugs. Therefore, many ruthenium complexes have been designed and synthesized with several ligands, resulting in metallodrugs with action in cancer cells of different histological types. Interestingly, this feature is strictly dependent on the nature of the ligands, and the complexes can act via diverse mechanisms, including induction of reactive oxygen species (ROS), binding of DNA, and cell death via apoptosis pathways [2,3,4,5,6,7,8,9,10]. In particular, NAMI-A ([ImH][trans-RuCl_4_(DMSO)(Im)], where Im = imidazole and DMSO = dimethylsulfoxide) and KP1019 ([IndH][trans-RuCl_4_(Ind)_2_], where Ind = indazole) are currently in phase I/II clinical trials, with promising results [11,12].

**Figure 1:**
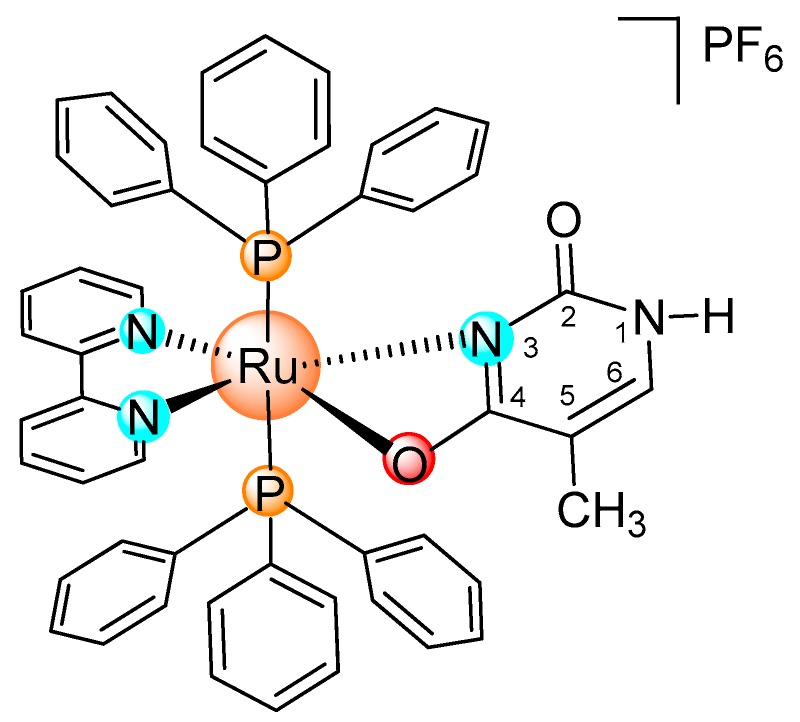
Chemical structure of the [Ru(PPh_3_)_2_(Thy)(bipy)]PF_6_ complex.

In our previous study, we designed, synthesized, and identified the Ru(II)-thymine complex [Ru(PPh_3_)_2_(Thy)(bipy)]PF_6_ (where PPh = triphenylphosphine, Thy = thymine and bipy = 2,2′-bipyridine) as a potent cytotoxic agent with the ability to bind DNA and human and bovine serum albumins [13]; however, the mechanisms of action of this complex in cancer cells have not been clearly demonstrated. In this study, the mechanism that is underlying the cytotoxicity of the [Ru(PPh_3_)_2_(Thy)(bipy)]PF_6_ complex (Figure 1) was assessed in human promyelocytic leukemia HL-60 cells.

## 2. Results and Discussion

### 2.1. The [Ru(PPh_3_)_2_(Thy)(bipy)]PF_6_ Complex Exhibits Potent Cytotoxicity in Different Cancer Cells

The cytotoxicity of the [Ru(PPh_3_)_2_(Thy)(bipy)]PF_6_ complex in eight cancer cell lines, HL-60 (human promyelocytic leukemia), K-562 (human chronic myelogenous leukemia), HCT116 (human colon carcinoma), MCF7 (human breast carcinoma), HepG2 (human hepatocellular carcinoma), HSC-3 (human oral squamous cell carcinoma), SCC-9 (human oral squamous cell carcinoma), B16-F10 (mouse melanoma), two noncancerous cells, MRC-5 (human lung fibroblast), and PBMC (human peripheral blood mononuclear cell), was evaluated using the alamar blue (AB) assay after 72 h of incubation. This small panel of cancer cell lines is representative of colon, breast, liver, tongue, gastric, skin, and hematological cancers. Table 1 shows the IC_50_ (half maximal inhibitory concentration) values. The complex presented IC_50_ values ranging from 1.1 to 11.1 μM for the HSC-3 and MCF7 cancer cells, respectively. The IC_50_ values for noncancerous cells were 11.5 and 1.7 μM for the MRC-5 cells and PBMCs, respectively. Doxorubicin presented IC_50_ values that were ranging from 0.1 to 1.1 μM for the HCT116 and MCF7 cancer cells, respectively, and 1.5 and 5.1 μM for the noncancerous cells MRC-5 and PBMC, respectively. Oxaliplatin presented IC_50_ values ranging from 0.6 to 5.7 μM for the HL-60 and MCF7 cancer cells, respectively, and 1.3 and 9.4 μM for the noncancerous cells MRC-5 and PBMC. Table 2 displays the selectivity index (SI) that was calculated, showing that the complex has an equal or higher SI than the positive controls doxorubicin and oxaliplatin, for many types of cancer cells.

**Figure 2:**
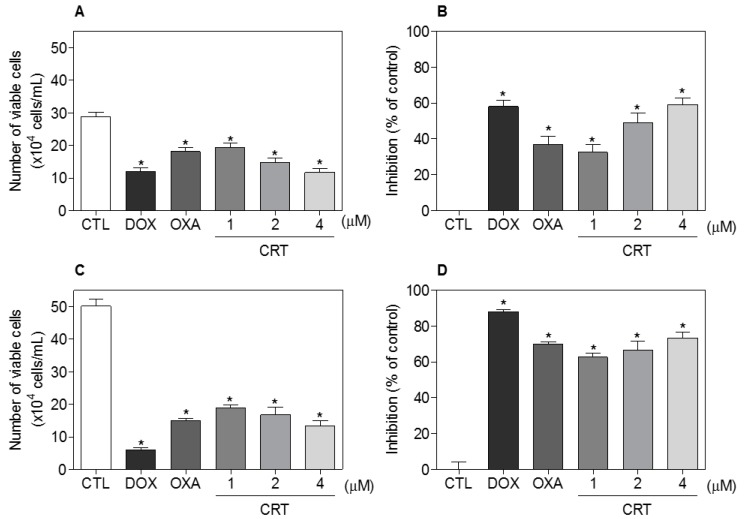
Effect of the [Ru(PPh_3_)_2_(Thy)(bipy)]PF_6_ complex (CRT) on the cell viability of HL-60 cells determined by the trypan blue exclusion (TBE) assay after 24 (**A**,**B**) and 48 (**C**,**D**) h of incubation. The negative control (CTL) received 0.1% DMSO, and the positive controls received doxorubicin (DOX, 2 µM) or oxaliplatin (OXA, 2.5 µM). Data are presented as the mean ± S.E.M. of three independent experiments performed in duplicate. * *p* < 0.05 as compared with the negative control by ANOVA, followed by the Student Newman-Keuls test.

In a new set of experiments, the human promyelocytic leukemia HL-60 cell line was used as a cellular model, since this cell line was among the most sensitive to the [Ru(PPh_3_)_2_(Thy)(bipy)]PF_6_ complex. Moreover, the HL-60 cell line is a cellular model often used to study the mechanism of the antileukemia action of new compounds [14,15,16,17,18]. Cell viability after treatment with the complex was confirmed by a trypan blue exclusion (TBE) assay in HL-60 cells after 24 and 48 h of incubation (Figure 2). At concentrations of 1, 2, and 4 μM, the complex reduced the number of viable cells by, respectively, 32.5%, 49.1%, and 59.1% after 24 h and by 62.6%, 66.6% and 73.4% after 48 h. No significant increase in the number of nonviable cells was observed. Doxorubicin (2 μM) reduced the number of viable cells by 58.0% after 24 h and by 88.1% after 48 h, while oxaliplatin (2.5 μM) reduced the number of viable cells by 37.0% after 24 h and by 70.0% after 48 h.

Other ruthenium complexes have been previously reported as potent cytotoxic agents, including cyclometalated ruthenium β-carboline complexes, which were cytotoxic to lung, liver, breast, and cervical cancers [8]; piplartine-containing ruthenium complexes, which were cytotoxic to colon, tongue, liver, breast, skin, and hematological cancers [5]; a ruthenium complex with xanthoxylin, which was cytotoxic to colon, breast, liver, tongue, gastric, skin, and hematological cancers [9]; ruthenium imidazole complexes, which were cytotoxic to lung, liver, breast, and cervical cancers [19]; and, a ruthenium-based 5-fluorouracil complex, which had enhanced cytotoxicity to breast, colon, liver, tongue, skin, and hematological cancers [10]. The IC_50_ values of these compounds are below 10 μM for most of the tested cancer cell lines. Herein, the Ru(II)-thymine complex presented IC_50_ values below 3 μM for most of the tested cancer cell lines. These data corroborate our previous study, where this complex was tested against a small panel of cancer cells (B16-F10, HepG2, K562, and HL-60), with which it had IC_50_ values below 2 μM [13].

### 2.2. The [Ru(PPh_3_)_2_(Thy)(bipy)]PF_6_ Complex Triggers Caspase-Mediated Apoptosis in HL-60 Cells

**Figure 3:**
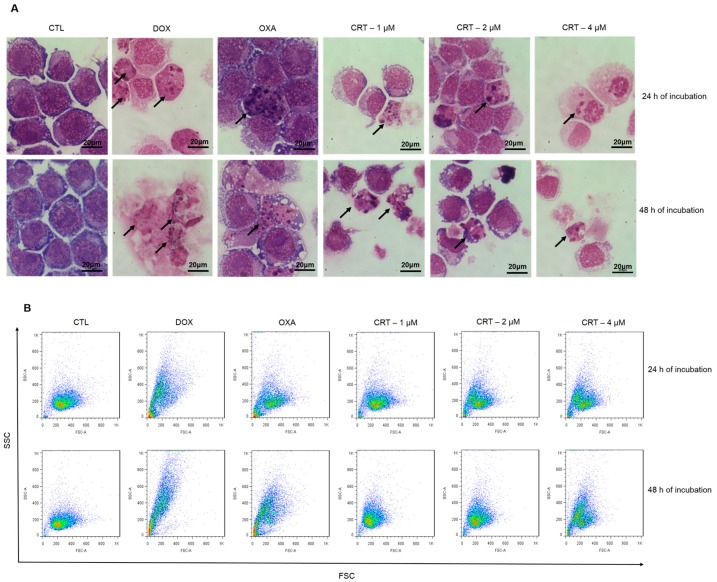
Effect of the [Ru(PPh_3_)_2_(Thy)(bipy)]PF_6_ complex (CRT) on the morphology of HL-60 cells after 24 and 48 h of incubation. (**A**) Cells stained with May-Grunwald-Giemsa and were examined by light microscopy (bar = 20 µm). Arrows indicate cells with reduced cell volume, chromatin condensation or fragmented DNA. (**B**) Light scattering features determined by flow cytometry. The negative control (CTL) received 0.1% DMSO, and the positive controls received doxorubicin (DOX, 2 µM) or oxaliplatin (OXA, 2.5 µM). The dot plots are expressed in arbitrary units. FSC: forward scatter; SCC: side scatter.

**Figure 4:**
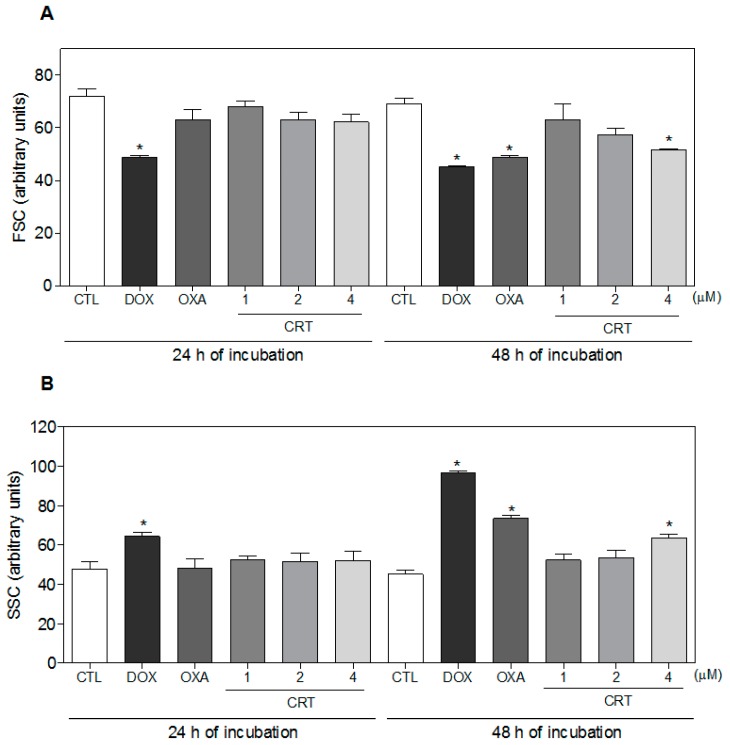
Effect of the [Ru(PPh_3_)_2_(Thy)(bipy)]PF_6_ complex (CRT) on the morphology of HL-60 cells after 24 and 48 h of incubation. (**A**) Quantification of forward light scatter (FSC) determined by flow cytometry; and (**B**) Quantification of side scatter (SCC), as determined by flow cytometry. The negative control (CTL) received 0.1% DMSO, and the positive controls received doxorubicin (DOX, 2 µM) or oxaliplatin (OXA, 2.5 µM). Data are presented as the mean ± S.E.M. of at the least three independent experiments. * *p* < 0.05 as compared with the negative control by ANOVA, followed by the Student Newman-Keuls test.

The biochemical and morphological correlates of apoptotic cell death include phosphatidylserine exposure, loss of the mitochondrial transmembrane potential (intrinsic apoptosis), activation of caspases, DNA fragmentation (karyorrhexis), chromatin condensation (pyknosis), cytoplasmic shrinkage, dynamic membrane blebbing, and the formation of apoptotic bodies [20,21]. HL-60 cells that were treated with the [Ru(PPh_3_)_2_(Thy)(bipy)]PF_6_ complex showed cell morphology changes that were associated with apoptosis, including a reduction in the cell volume, chromatin condensation, and fragmentation of the nuclei, as observed in May-Grunwald-Giemsa-stained cells (Figure 3A). Furthermore, the complex caused cell shrinkage, as indicated by the decrease in forward light scatter (FSC) (Figure 3B and Figure 4A), as well as nuclear condensation, as indicated by an increase in side scatter (SCC) (Figure 3B and Figure 4B), which were both assessed by flow cytometry. Doxorubicin and oxaliplatin also caused cell death by apoptosis.

The internucleosomal DNA fragmentation and cell cycle distribution were assessed in HL-60 cells after 24 and 48 h of incubation with the [Ru(PPh_3_)_2_(Thy)(bipy)]PF_6_ complex in a DNA content-based assay using the dye propidium iodide (PI) and flow cytometry (Table 3). All DNA with a subdiploid size (sub-G_0_/G_1_) were considered to be fragmented. At concentrations of 1, 2, and 4 μM, the complex led to, respectively, 19.4%, 30.1%, and 36.2% DNA fragmentation after 24 h of incubation and to 12.5%, 26.7%, and 58.2% DNA fragmentation after 48 h of incubation. Doxorubicin also induced DNA fragmentation. Oxaliplatin caused cell cycle arrest at the G_2_/M phase and induced DNA fragmentation.

**Figure 5:**
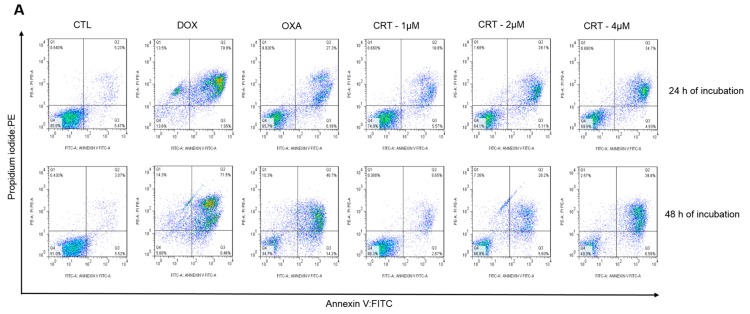

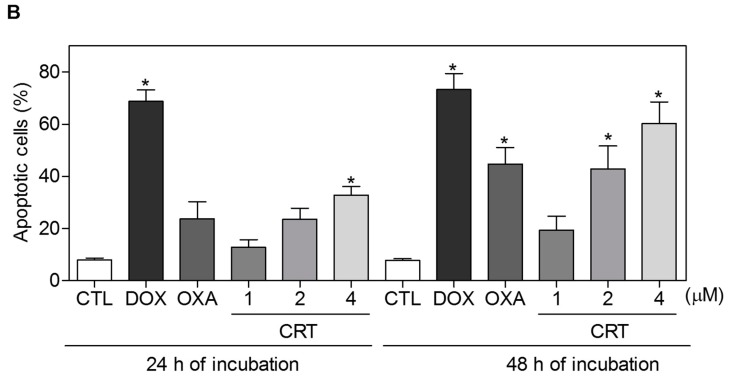
Evaluation of the apoptosis induced by the [Ru(PPh_3_)_2_(Thy)(bipy)]PF_6_ complex (CRT) in HL-60 cells determined by flow cytometry using annexin V-FITC/PI staining after 24 and 48 h of incubation. (**A**) Representative flow cytometry dot plots. (**B**) Apoptotic cell quantification (annexin V-FITC positive cells). The negative control (CTL) received 0.1% DMSO, and the positive controls received doxorubicin (DOX, 2 µM) or oxaliplatin (OXA, 2.5 µM). Data are presented as the mean ± S.E.M. of at the least three independent experiments. * *p* < 0.05 as compared with the negative control by ANOVA, followed by the Student Newman-Keuls test.

Additionally, annexin V, which is a Ca^2+^-dependent protein with high affinity for phosphatidylserine, was conjugated to a fluorochrome to detect phosphatidylserine exposure using flow cytometry. Since phosphatidylserine exposure precedes the loss of membrane integrity, annexin V staining was used along with the dye PI to identify early and late apoptotic cells, as well as necrotic cells [22]. Therefore, annexin V-FITC/PI double staining was applied to HL-60 cells that were treated with the [Ru(PPh_3_)_2_(Thy)(bipy)]PF_6_ complex after 24 and 48 h of incubation, and the numbers of viable, early apoptotic, late apoptotic, and necrotic cells were quantified by flow cytometry (Figure 5). The complex induced a significant increase in the percentage of apoptotic cells (early apoptotic cells + late apoptotic cells). No significant increase in the percentage of necrotic cells was observed.

**Figure 6:**
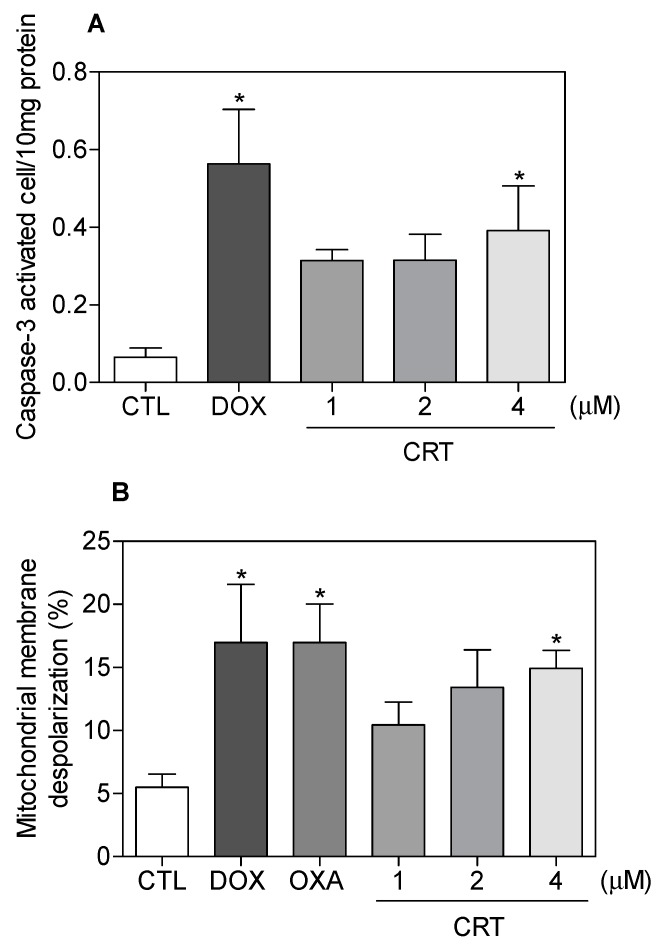
Caspase-3 activity (**A**), assessed by a colorimetric assay after 48 h of incubation, and mitochondrial membrane potential (**B**), evaluated by flow cytometry using rhodamine 123 staining after 24 h of incubation, of HL-60 cells treatment with the [Ru(PPh_3_)_2_(Thy)(bipy)]PF_6_ complex (CRT). The negative control (CTL) received 0.1% DMSO, and the positive controls received doxorubicin (DOX, 2 µM) or oxaliplatin (OXA, 2.5 µM). Data are presented as the mean ± S.E.M. of at the least three independent experiments. * *p* < 0.05 as compared with the negative control by ANOVA, followed by the Student Newman-Keuls test.

**Figure 7:**
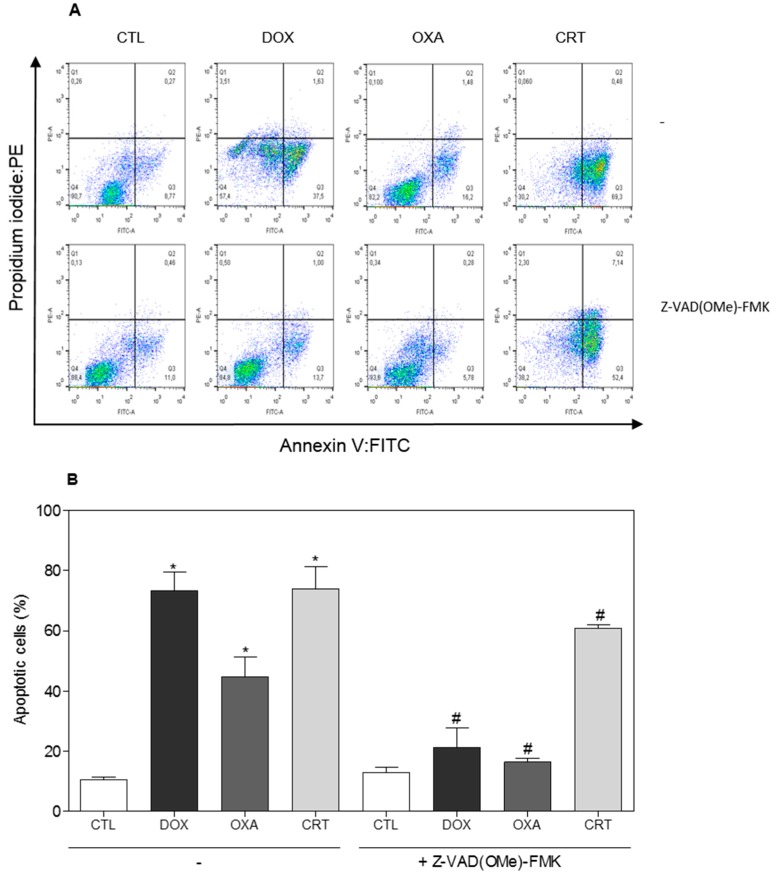
Prevention of the apoptosis induced by the [Ru(PPh_3_)_2_(Thy)(bipy)]PF_6_ complex (CRT) in HL-60 cells by a pan-caspase inhibitor (Z-VAD(OMe)-FMK), as assessed by flow cytometry using annexin V-FITC/PI staining. (**A**) Representative flow cytometry dot plots. (**B**) Apoptotic cell quantification (annexin V-FITC positive cells). The cells were pretreated for 2 h with 50 µM Z-VAD(OMe)-FMK and then incubated with 4 μM CRT for 48 h. The negative control (CTL) received 0.1% DMSO, and the positive controls received doxorubicin (DOX, 2 µM) or oxaliplatin (OXA, 2.5 µM). Data are presented as the mean ± S.E.M. of at the least three independent experiments. * *p* < 0.05 as compared with the negative control by ANOVA, followed by the Student Newman-Keuls test. ^#^ *p* < 0.05 as compared with the respective treatment without inhibitor by ANOVA, followed by the Student Newman-Keuls test.

Loss of the mitochondrial transmembrane potential is a critical step in intrinsic apoptosis, and activation of caspase-3 (an executioner caspase) favors DNA fragmentation [20,21]. The complex also induced activation of caspase-3 in HL-60 cells (Figure 6A) and it caused the loss of the mitochondrial transmembrane potential (Figure 6B). In addition, cotreatment with a pan-caspase inhibitor, Z-VAD(OMe)-FMK, partially prevented the complex-induced increase in apoptotic cells (Figure 7), indicating the induction of caspase-mediated apoptosis in HL-60 cells by the [Ru(PPh_3_)_2_(Thy)(bipy)]PF_6_ complex. On the other hand, additional mechanisms also appear to be involved in the complex induced-apoptosis, since the pan-caspase inhibitor was not able to fully inhibit the apoptosis that was caused by the complex. Doxorubicin and oxaliplatin also caused an increase in the percentage of apoptotic cells.

As previously mentioned, many ruthenium complexes are known to induce apoptosis. Ruthenium imidazole complexes induce G_0_/G_1_ arrest and apoptosis in A549 and NCI-H460 cells through extrinsic and intrinsic mitochondrial pathways. In addition, these complexes induce cytoprotective autophagy via the ROS-mediated extracellular signal–regulated kinase (ERK) signaling pathway, and the inhibition of autophagy could facilitate cell apoptosis [19]. The ruthenium complex with xanthoxylin induces S-phase arrest and causes ERK1/2-mediated apoptosis in HepG2 cells through a p53-independent pathway [9]. The ruthenium complexes with phenylterpyridine derivatives trigger death receptor-mediated apoptosis in A375 cells [23]. Cyclometalated ruthenium β-carboline complexes induce apoptosis in HeLa cells, mainly through mitochondrial dysfunction, intracellular ROS accumulation, and ROS-mediated DNA damage [8]. Piplartine-containing ruthenium complexes induce caspase-dependent and intrinsic mitochondrial apoptosis in HCT116 cells through a ROS-mediated pathway [5]. Ruthenium polypyridyl complexes induce apoptosis in BEL-7402 cells through a ROS-mediated mitochondrial dysfunction pathway [24]. Moreover, the ruthenium-based 5-fluorouracil complex induces caspase-mediated apoptosis in HCT116 cells [10]. Herein, we observed that the Ru(II)-thymine complex induces caspase-mediated apoptosis in HL-60 cells.

The protein Bcl-2-associated death promoter (BAD) is a pro-apoptotic member of the Bcl-2 family [20,21]. The cytotoxicity of the [Ru(PPh_3_)_2_(Thy)(bipy)]PF_6_ complex was also examined in BAD gene knockout immortalized mouse embryonic fibroblasts (BAD KO SV40 MEF) and its parental cell line, wild-type immortalized mouse embryonic fibroblasts (WT SV40 MEF) while using the AB assay after 72 h of incubation. The complex presented IC_50_ values of 2.4 μM in BAD KO SV40 MEF cells and 2.2 μM in WT SV40 MEF cells, suggesting that the BAD gene is not essential for the cytotoxicity that is induced by the tested complex. Doxorubicin presented IC_50_ values of 1.3 and 0.8 μM, and 5-fluorouracil presented IC_50_ values of 11.2 and 5.7 μM in BAD KO SV40 MEF and WT SV40 MEF cell, respectively.

The induction of ROS production is also an important mechanism for inducing apoptosis. However, the [Ru(PPh_3_)_2_(Thy)(bipy)]PF_6_ complex did not induce a significant increase in ROS levels after 1 or 3 h of incubation at the tested concentrations (data not shown). Moreover, pretreatment with the antioxidant *N*-acetyl-l-cysteine (NAC) did not prevent the increase in apoptotic cells that was caused by the complex (data not shown).

In conclusion, the [Ru(PPh_3_)_2_(Thy)(bipy)]PF_6_ complex displays potent cytotoxicity to different cancer cells. In studies of the underlying cytotoxic mechanism, we observed the morphology associated with apoptotic cell death, increased internucleosomal DNA fragmentation without cell membrane permeability changes, a loss of the mitochondrial transmembrane potential, increased phosphatidylserine externalization, and caspase-3 activation in complex-treated HL-60 cells. The pretreatment of HL-60 cells with Z-VAD(OMe)-FMK, which is a pan-caspase inhibitor, partially reduced the apoptosis that was induced by the complex, indicating that the apoptotic cell death occurred through caspase-mediated pathways.

## 3. Materials and Methods

### 3.1. Synthesis of the [Ru(PPh_3_)_2_(Thy)(bipy)]PF_6_ Complex

The [Ru(PPh_3_)_2_(Thy)(bipy)]PF_6_ complex was obtained, as previously described by Correa et al. [13]. Briefly, thymine (23 mg; 0.18 mmol) was dissolved in a Schlenk flask with 50 mL of a dichloromethane/methanol mixture (1:1 *v*/*v*) containing triethylamine (10 μL) and KPF 6 (0.12 mmol; 15.0 mg). Next, 100 mg (0.12 mmol) of the precursor [RuCl_2_(PPh_3_)_2_(bipy)] was added. The solution was kept under reflux and in an inert atmosphere, and was stirred for 48 h. The final solution was concentrated to 2 mL, and 10 mL of water was added to precipitate an orange powder. The solids were filtered off, washed with separately with warm water and diethyl ether, and then dried under vacuum. All of the manipulations were performed under argon. All of the reagents were purchased from Sigma-Aldrich (Sigma-Aldrich Co., Saint Louis, MO, USA) and were used as received.

### 3.2. Cells and Cytotoxicity Assay

The cell lines that were used in this study were obtained from the American Type Culture Collection (ATCC) and were cultured, as described previously [25]. Primary PBMCs were obtained with informed consent (# 031019/2013). All the cells were free of contamination, and the cell viability was determined by the TBE or AB assay, which were performed following the procedure that was described previously [25,26]. The cell morphology analysis was performed using cytospin and May-Grunwald-Giemsa staining as described previously [25]. To investigate caspase-3 activation, a caspase-3 colorimetric assay kit (Sigma-Aldrich Co.) was used.

### 3.3. Flow Cytometric Assays

Light scattering features were determined by flow cytometry. The internucleosomal DNA fragmentation and cell cycle distribution were determined according to Nicoletti et al. [27]. For apoptosis measurements, a FITC Annexin V Apoptosis Detection Kit I (BD Biosciences, San Jose, CA, USA) was used, and the analysis was performed, according to the manufacturer’s instructions. A protection assay using the pan-caspase inhibitor Z-VAD(OMe)-FMK (Cayman Chemical, Ann Arbor, MI, USA) was also evaluated. The mitochondrial transmembrane potential was determined by the retention of the dye rhodamine 123 [28]. The levels of ROS were measured, as previously described [29], using 2′,7′-dichlorofluorescin diacetate (DCF-DA, Sigma-Aldrich Co.). At least 1 × 10^4^ events were recorded per sample using a BD LSRFortessa cytometer along with BD FACSDiva Software (BD Biosciences) and Flowjo Software 10 (Flowjo LCC, Ashland, OR, USA). The cellular debris was omitted from the analysis.

### 3.4. Statistical Analysis

Data were analyzed and graphs were generated using GraphPad Prism software to evaluate significance. Differences among experimental groups were compared using analysis of variance (ANOVA), followed by the Student–Newman–Keuls test (*p* < 0.05).

## Figures and Tables

**Table 1:** Cytotoxicity of the [Ru(PPh_3_)_2_(Thy)(bipy)]PF_6_ complex (CRT).

Cells	Histological Type	IC_50_ in µM		
		DOX	OXA	CRT
Cancer cells				
HL-60	Human promyelocytic leukemia	0.3	0.6	1.4
		0.3–0.4	0.1–0.8	0.7–2.8
K562	Human chronic myelogenous leukemia	0.3	1.0	1.3
		0.2–0.5	0.1–1.3	0.9–2.0
HCT116	Human colon carcinoma	0.1	4.1	1.6
		0.1–0.2	2.7–6.4	1.2–2.2
MCF7	Human breast carcinoma	1.1	5.7	11.1
		0.3–3.5	3.3–9.4	7.9–15.5
HepG2	Human hepatocellular carcinoma	0.1	2.2	2.8
		0.1–0.2	1.3–3.8	2.3–3.3
HSC3	Human oral squamous cell carcinoma	0.3	3.1	1.1
		0.2–0.4	1.6–5.3	0.8–1.5
SCC9	Human oral squamous cell carcinoma	0.5	N.d.	2.4
		0.4–0.7		1.7–3.5
B16-F10	Mouse melanoma	0.1	2.2	1.4
		0.1–0.2	1.2–4.1	1.2–1.8
Non-cancer cells				
MRC5	Human lung fibroblast	1.5	1.3	11.5
		1.2–2.0	1.0–2.2	10.3–12.9
PBMC	Human peripheral blood mononuclear cells	5.1	9.4	1.7
		3.2–8.2	6.5–11.4	1.4–2.0

Data are presented as IC_50_ values in μM and their respective 95% confidence interval, obtained by nonlinear regression from at the least three independent experiments, measured by the alamar blue (AB) assay after 72 h of incubation. Doxorubicin (DOX) and oxaliplatin (OXA) were used as the positive controls. N.d. Not determined.

**Table 2:** Selectivity index of the [Ru(PPh_3_)_2_(Thy)(bipy)]PF_6_ complex (CRT).

Cancer Cells	Non-Cancer Cells					
	MRC5			PBMC		
	DOX	OXA	CRT	DOX	OXA	CRT
HL-60	5	2.2	8.2	17	15.7	1.2
K-562	5	1.3	8.8	17	9.4	1.3
HCT116	15	0.3	7.2	51	2.3	1.1
MCF7	1.4	0.2	1	4.6	1.7	0.2
HepG2	15	0.6	4.1	51	4.3	0.6
HSC-3	5	0.4	10.5	17	3.0	1.6
SCC-9	3	N.d.	4.8	10.2	N.d.	0.7
B16-F10	15	0.6	8.2	51	4.3	1.2

Data are presented the selectivity index (SI) calculated using the following formula: SI = IC_50_[noncancerous cells]/IC_50_[cancer cells]. Cancer cells: HL-60 (human promyelocytic leukemia); K-562 (human chronic myelogenous leukemia); HCT116 (human colon carcinoma); MCF7 (human breast carcinoma); HepG2 (human hepatocellular carcinoma); HSC-3 (human oral squamous cell carcinoma); SCC-9 (human oral squamous cell carcinoma); and, B16-F10 (mouse melanoma). Noncancerous cells: MRC-5 (human lung fibroblast) and PBMC (human peripheral blood mononuclear cell). Doxorubicin (DOX) and oxaliplatin (OXA) were used as the positive controls. N.d. Not determined.

**Table 3:** Effect of the [Ru(PPh_3_)_2_(Thy)(bipy)]PF_6_ complex (CRT) on the cell cycle distribution of HL-60 cells.

Treatment	Concentration (µM)	DNA Content (%)			
		Sub-G_0_/G_1_	G_0_/G_1_	S	G_2_/M
24 h of incubation					
CTL	-	11.5 ± 1.4	50.7 ± 5.5	15.0 ± 2.5	16.3 ± 0.7
DOX	2	58.6 ± 2.5 *	19.9 ± 2.5 *	6.6 ± 1.6 *	8.9 ± 1.5 *
OXA	2.5	21.9 ± 3.3 *	31.4 ± 3.0 *	11.6 ± 2.3	26.0 ± 3.6 *
CRT	1	19.4 ± 3.9 *	30.5 ± 4.8 *	11.5 ± 1.7	18.5 ± 0.7
	2	30.1 ± 7.9 *	38.7 ± 2.2 *	7.8 ± 1.5 *	17.9 ± 4.4
	4	36.2 ± 9.5 *	36.3 ± 3.1 *	10.4 ± 2.4	13.2 ± 4.1
48 h of incubation					
CTL	-	7.5 ± 1.0	58.5 ± 3.1	14.1 ± 1.3	17.0 ± 1.6
DOX	2	65.4 ± 2.8 *	21.9 ± 3.0 *	6.5 ± 0.6 *	3.8 ± 0.9 *
OXA	2.5	18.4 ± 4.4 *	42.9 ± 2.8 *	14.2 ± 1.2	19.1 ± 3.3
CRT	1	12.5 ± 3.5 *	41.2 ± 9.6 *	11.2 ± 2.4	10.8 ± 2.5
	2	26.7 ± 7.0 *	42.6 ± 4.9 *	10.5 ± 0.8 *	14.4 ± 2.5
	4	58.2 ± 8.8 *	28.4 ± 5.9 *	4.4 ± 1.2 *	5.5 ± 2.6 *

Data are presented as the mean ± S.E.M. of three independent experiments performed in duplicate. The negative control (CTL) received 0.1% DMSO, and the positive controls received doxorubicin (DOX, 2 µM) or oxaliplatin (OXA, 2.5 µM). * *p* < 0.05 compared with the negative control by ANOVA, followed by Student Newman-Keuls test.
